# Intervention-induced changes in state mindfulness do not predict trait changes in mindfulness, self-compassion, or perceived stress

**DOI:** 10.1038/s41598-025-27697-0

**Published:** 2025-11-21

**Authors:** Kira S. A. Borgdorf, Gabriela Küchler, Cornelia Wrzus, Corina Aguilar-Raab

**Affiliations:** 1 Department of Clinical Psychology, Interaction- and Psychotherapy Research, Institute for Compassionate Awareness and Interdependence Research and Practice IN‐CARE, University of Mannheim, Mannheim, Germany; 2 Psychological Institute, Heidelberg University, Heidelberg, Germany; 3 Network Aging Research, Heidelberg University, Heidelberg, Germany; 4 Institute for Medical Psychology, University Hospital Heidelberg, Heidelberg, Germany

**Keywords:** Mindfulness, Self-compassion, Perceived stress, State-trait associations, Socioemotional intervention, Psychology, Quality of life

## Abstract

Previous research has identified state mindfulness as a key change mechanism in explicitly mindfulness-based interventions to increase trait mindfulness and reduce stress. This study extends previous work by examining trajectories of state mindfulness and their associations with trait indicators of mindfulness, self-compassion, and perceived stress when mindfulness practices are delivered within a Socioemotional Competence Training (SECT) without explicit mindfulness labeling. A sample of 166 healthy younger and older German adults (75.2% female; *M*_age_ = 46.26; *SD*_age_ = 18.70) participated in the 8-week SECT. State mindfulness was assessed weekly, whereas trait mindfulness, self-compassion, and perceived stress were assessed before, during, and after the training. Second-order latent growth models suggest significant pre-post improvements in all state and trait variables (*d* = |0.38| to |0.62|), but trait changes were not predicted by changes in state mindfulness. These findings indicate that while state mindfulness increases during the Socioemotional Competence Training, these increases alone appear insufficient to drive changes in trait mindfulness and related outcomes. Results suggest that intervention framing could play a crucial role in facilitating explicit connections between state experiences and trait development, contributing to our understanding of mindfulness as a change mechanism in psychological interventions.

**Supplementary Information:**

The online version contains supplementary material available at 10.1038/s41598-025-27697-0.

## Introduction

The concept of mindfulness and mindfulness practices has seen an impressive surge in scientific interest over the last three decades^1^. Mindfulness is generally defined as a purposeful present-moment awareness with a non-judgmental and accepting attitude^1–5^. Research has approached mindfulness as both a relatively stable *trait*-like characteristic and a fluctuating *state *experience. Mindfulness construed as a trait-like characteristic has been defined, and mostly measured with self-report questionnaires, as a general tendency or predisposition to be attentive and non-judgmental towards present-moment experiences (e.g^3,5–11^). The *momentary* non-judgmental orientation to present-moment experiences is referred to as state mindfulness^12–15^. Ecological momentary assessment studies suggest that state mindfulness naturally varies in everyday life and between individuals^15,16^.

Extensive research demonstrates that mindfulness-based interventions (MBIs) yield numerous positive outcomes, including improved emotion and behavioral regulation, cognitive functioning, increased well-being, better physical health indicators, and more prosocial attitudes^4,17–32^; for a discussion of boundary conditions, see^33^). While these benefits are well-documented, researchers continue investigating the underlying change mechanisms. Similar to psychotherapy^34^ and personality interventions^35^, MBIs are hypothesized to operate through both common and specific change mechanisms (e.g^24,25,36,37^). Common change factors include therapeutic alliance, group cohesion, and expectation of beneficial outcomes (e.g^37^). Specific mechanisms encompass state mindfulness, emotion regulation, interoceptive awareness, cognitive processing changes, and shifts in self-perspective (e.g^11,38^). Most relevant to the current investigation, repeated experiences of state mindfulness are thought to facilitate cognitive and emotional meta-awareness, allowing for more conscious selection of evaluative information and less automatic, reactive responses to internal and external experiences^36,39^.

To understand how these mechanisms work, or more specifically, how state experiences relate to lasting trait change during interventions, researchers have suggested several theoretical frameworks. By applying ideas from Whole Trait Theory (WTT^40^;, scholars have aimed to reconcile the state and trait approach to mindfulness by conceptualizing trait mindfulness as a stable density distribution of fluctuating, within-person mindfulness state experiences^15^. WTT conceptualizes traits as consisting of two interactively emerging parts: (a) as descriptions of how individuals typically function (descriptive part; “density distributions of states”) as well as (b) as explanations of how and why trait expression differs across individuals, contexts, and time (explanatory part). For example, the descriptive component of trait mindfulness captures how often someone typically experiences mindful states. The explanatory component examines differences in socio-cognitive mechanisms, such as goals, motivations, or contextual factors, that may account for different mindful state expressions in different situations^15,40^. This perspective aligns with broader theories of personality development and psychotherapy efficacy, which emphasize that repeated state experiences can accumulate into trait-level changes over time (e.g^41,42^). Empirical findings corroborate these theoretical ideas, showing, for example, that weekly changes in personality characteristics correlate with personality trait change in app-based and in-person interventions^43,44^. Applying the tenets of WTT to mindfulness, Warren and colleagues^15^ suggest that the frequency and duration of mindful state experiences constitute trait mindfulness. Consequently, if individuals experience mindful states more frequently—for example, during MBIs—indicators of their trait mindfulness should change accordingly^3,15^.

Consistent with these theoretical assumptions, recent studies incorporating measures of state mindfulness have provided first evidence for the outlined state-trait associations (e.g^45–48^). Two studies examining state trajectories across 8-week Mindfulness-Based Stress Reduction (MBSR) courses found that weekly increases in state mindfulness predicted pre-post changes in trait mindfulness and psychological stress indicators^46,48^. These findings underscore that state-trait mechanisms may be crucial for how MBIs create lasting trait change^15,39^ and aligns with the understanding that mindfulness is trainable through repeated practice^4^.

Interestingly, meta-analytic evidence suggests that interventions not explicitly focusing on mindfulness also yield increases in mindfulness, although to a smaller extent than MBIs (^49^; cf^30,31^.). This includes mindfulness-*informed* interventions, such as Acceptance and Commitment Therapy (ACT) or Dialectical Behavior Therapy (DBT), which explicitly integrate mindfulness practices but embed them within a broader set of techniques, such as skills training, cognitive restructuring, and values clarification^50,51^. These approaches differ from MBIs in that mindfulness is not their sole theoretical foundation. Research shows that they still increase trait mindfulness and reduce symptoms of anxiety and depression^52–55^. More surprisingly, increases in trait mindfulness have also been observed in interventions where mindfulness practices are typically absent. Notably, one meta-analysis compared traditional MBIs (e.g., MBSR and Mindfulness-Based Cognitive Therapy, explicitly excluding mindfulness-informed interventions) to non-mindfulness-based active bona fide conditions intended to be therapeutic (e.g., Cognitive Behavioral Therapy [CBT]) on trait mindfulness and clinical outcomes, such as depression, anxiety, and psychological distress^49^. These comparisons are especially informative because they control for non-specific and specific treatment factors (e.g., cognitive restructuring as in CBT) simultaneously.

Taken together, these findings suggest that mindfulness may be cultivated explicitly (MBIs), secondarily (mindfulness-informed interventions), or implicitly in interventions without mindfulness content, such as CBT (e.g., through observation of thoughts and emotions^24,31,37,49^). The open question remains whether the state-trait pathway found previously^46,48^ generalizes beyond explicit MBIs to contexts where mindfulness components are embedded more secondarily or implicitly within broader intervention frameworks (cf^36,56^. We aimed to shed light on this question in two ways: First, by replicating previous study results on mindfulness and perceived stress but in a training context without explicit mindfulness reference, namely during an 8-week Socioemotional Competence Training (SECT). The training was designed for healthy adults seeking to improve stress management and social relationships and included various mindfulness exercises without referring to them as such. Thus, the SECT can be situated conceptually between interventions without any mindfulness reference (e.g., CBT), and mindfulness-informed interventions (e.g., ACT, DBT). Second, we examined whether the state–trait pathway generalizes to a broader range of outcomes.

Perceived stress is particularly relevant because stress reduction is one of the most frequent targets of psychological interventions in general, and MBIs in particular^5,57,58^. Although the experience of stress varies situationally, individuals show stable differences in their general tendency to perceive and appraise situations as stressful, which suggests that perceived stress can be conceptualized as a trait^59,60^. Like other traits^61^, trait perceived stress is malleable, with mindfulness interventions showing strong effects on these stress indicators^37^. As mentioned earlier, previous research has demonstrated that weekly increases in mindfulness predicted pre-post reductions in trait stress indicators^46,48^. We therefore examined whether such state-trait associations would replicate in a socioemotional competence training without explicit mindfulness reference.

Self-compassion—treating oneself with kindness when facing difficulty—shares conceptual overlap with mindfulness, as mindful awareness of difficult experience is an integral part of self-compassion’s definition^62,63^. Substantial empirical evidence demonstrates bidirectional relationships between these constructs: Some researchers identified self-compassion as a change mechanism in MBIs^24,64,65^, while others consider mindfulness as a prerequisite for self-compassion to arise^62,63,66–68^. Still others have found a combination of both to mediate effects on well-being^69^. Hence, the relationship between mindfulness and self-compassion provides a particularly compelling context for examining state mindfulness mechanisms. In the current study, we included self-compassion as an additional outcome to further elucidate on the assumed direction that mindful awareness is a precondition for self-compassion to arise (cf.^67^).

### Present research

The goal of the current research was twofold: First, we wanted to investigate whether the effects of state mindfulness extend beyond traditional MBIs to a Socioemotional Competence Training (SECT) that incorporated mindfulness practices without explicit mindfulness framing. This design allows examination of whether state mindfulness operates as a specific change mechanism while reducing expectancy effects associated with mindfulness labeling. And second, beyond replicating previous findings on trait mindfulness and perceived stress^46,48^, we included self-compassion as a potential “downstream” consequence of state mindfulness changes.

More specifically, we examined whether state mindfulness increases throughout the 8-week SECT^70^ and examined the relationships between weekly changes in state mindfulness and changes in trait mindfulness, perceived stress, and self-compassion. The SECT included some mindfulness practices (e.g., body scan, awareness of the breath, open monitoring) but was not designed, structured, or advertised as an MBI. Our preregistered hypotheses were:

#### Hypothesis 1

*(H1)*: Over the course of the training, state mindfulness improves.

#### Hypothesis 2

*(H2a)*: More pronounced changes in state mindfulness are associated with stronger changes in trait mindfulness.

#### Hypothesis

*(H2b)*: More pronounced changes in state mindfulness are associated with stronger changes in trait self-compassion.

#### Hypothesis

*(H2c)*: More pronounced changes in state mindfulness are associated with stronger changes in trait perceived stress.

Specifically, with increasing state mindfulness, we expected increases in trait mindfulness and self-compassion, and decreases in perceived stress, respectively. Moreover, because the sample was stratified into younger and older aged adults for a different research question of the general project^44^, we included age group as a moderator in all analyses. We did not have a priori hypotheses about age effects, but exploratory consideration of age differences may be informative given limited evidence in the literature: Preliminary findings suggest that older adults may experience modest benefits of MBIs in terms of psychological well-being and reduced distress, but overall intervention effectiveness in this growing population remains underexplored (e.g^71–73^). We therefore included age group as a control and exploratory moderator without specific expectations regarding its influence on the present hypotheses.

## Results

The SECT was conducted in an in-person format over 8 consecutive weeks and was carried out in three training cohorts, starting in January, April, and June 2023, respectively. The training focused on emotional stability (weeks 1–4: stress synthesis, resilience, attention- and emotion-regulation) and social skills (weeks 5–7: social dynamics, behavioral training), concluding with a wrap-up session in week 8 (for an overview of the training content see^70^. The study was designed as a randomized controlled trial, with two waitlist control groups participating in the training in the second or third cohort, respectively. State mindfulness was, however, only assessed during the active training period. Therefore, analyses focused on longitudinal changes. Participants completed self-report trait measures (mindfulness, self-compassion, and perceived stress) before, 4 weeks into, and after the training. State mindfulness was assessed weekly, approximately 5 days after each training module. Data were analyzed using multilevel models and second-order latent growth models to examine within-person and between-person effects and correlated change over time. Because the sample was stratified into younger and older adults, age group was included as a moderating factor in all analyses (see Method section for more details). Although we had no preregistered hypotheses about age effects for the present study, this inclusion both ensures statistical control and allows for exploratory insights.

### Sociodemographic information

In total, 166 adults (*M*_Age_ = 46.3, *SD*_Age_ = 18.7) participated in the training^1^, of whom 75.2% were female. A majority of the 81 younger (*M*_Age_ = 28.33, *SD*_Age_ = 4.92, range = 19–42 years) and 84 older adults (*n* = 84; *M*_Age_ = 63.55, *SD*_Age_ = 7.20, range 50–78 years) indicated holding a university degree as their highest educational achievement (63.0%). Almost all younger participants were either studying (55.6%) or employed (37.0%), whereas most older adults were either employed (46.4%) or retired (42.9%). Approximately half of the sample (50.3%) had prior meditation experience, of which 60.5% did not meditate at all or only infrequently (ranging from *not at all* to *once a month*), and 39.5% meditated at least *several times a month* to *daily*. Further sociodemographic information can be found in Supplementary Table S1. On average, the 166 participants completed 6.88 training sessions (*SD* = 0.89) and 7.18 weekly questionnaires (*SD* = 1.40). However, they did not provide information at all timepoints: In total, 160 participants filled out questionnaires at T1, 151 at T2, and 141 at T3. Participants who started in different cohorts did not differ on important baseline characteristics (all *p*s > 0.05). Moreover, participants who dropped out between T1 and T3 did not differ from those who completed the training (all *p*s > 0.05; see Supplementary Tables S2 and S3 as well as^70^ for further details).

### Increases in state and trait variables during the intervention

**Fig. 1:**
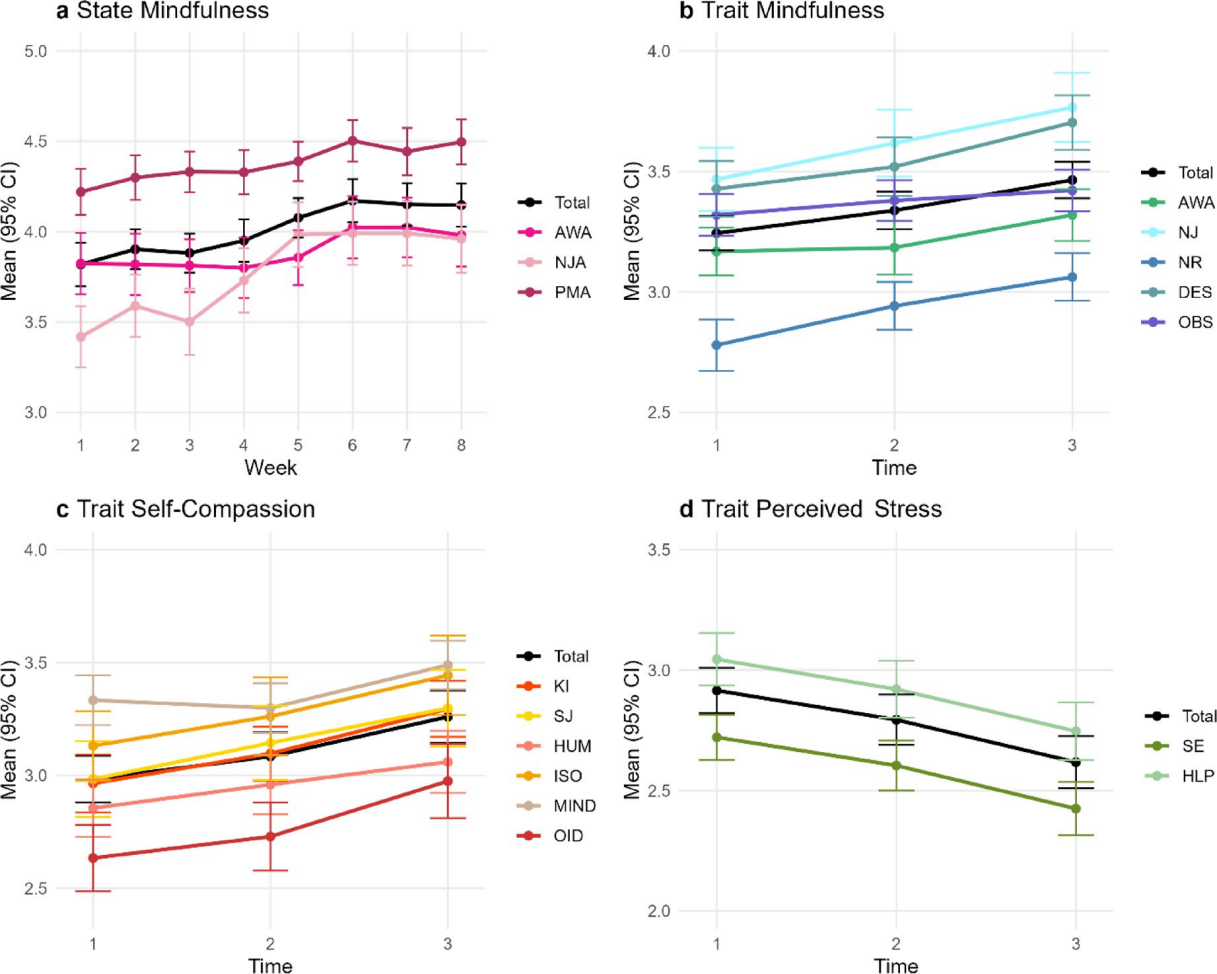
Mean Trajectories of State Mindfulness, Trait Mindfulness, Trait Self-Compassion, and Trait Perceived Stress. Trajectories of state mindfulness (**a**), trait mindfulness (**b**), trait self-compassion (**c**), and trait perceived stress (**d**), total mean scores and subscales with 95% Confidence Interval (CI). AWA = acting with awareness, NJA = nonjudgmental acceptance, PMA = present-moment attention; NJ = nonjudge; NR = nonreact; DES = describe; OBS = observe; KI = self-kindness; SJ = self-judgment; HUM= common humanity; ISO = isolation; MIND = mindfulness, OID = overidentification; SE = self-efficacy (reverse coded); HLP = helplessness.

Figure 1 shows the trajectories of state mindfulness, trait mindfulness, trait self-compassion, and trait perceived stress overall and on facet level. Descriptive statistics, reliabilities, and bivariate correlations are depicted in Supplementary Table S4.

State changes of mindfulness were analyzed with a multilevel model with time as a Level 1 random within-person predictor and age group (0 = younger, 1 = older adults) as a grand-mean centered Level 2 predictor. In line with H1, results indicate that state mindfulness significantly increased over time (*b *= 0.05, 95% CI [0.04; 0.07]). Age did not moderate this effect (*b *= −0.01, 95% CI [−0.03; 0.03]). 

**Fig. 2:**
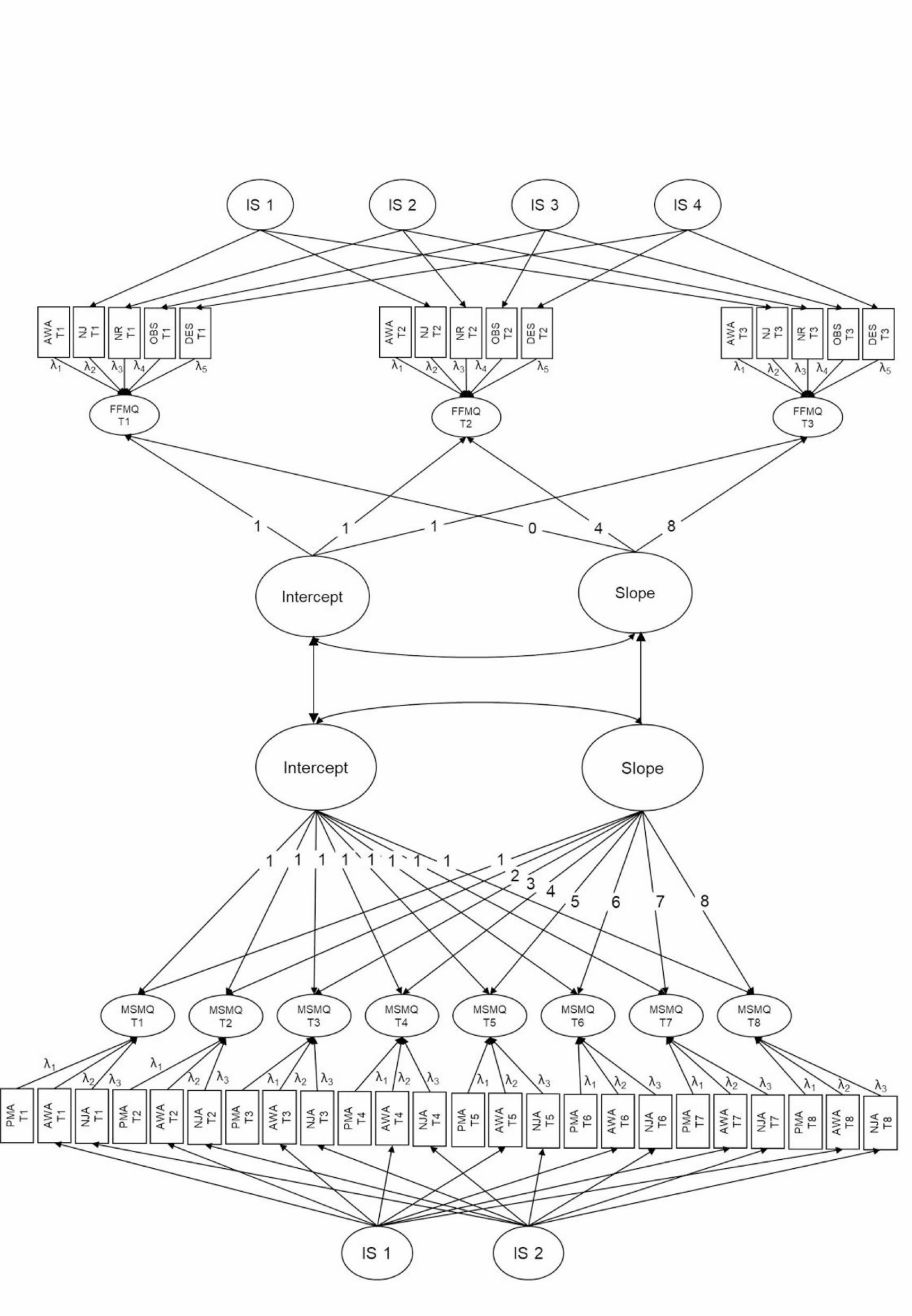
Second-Order Latent Growth Models: State Mindfulness Trajectories Predict Trait Mindfulness Change. Second-order latent growth model for state mindfulness (lower half; MSMQ) and trait mindfulness (upper half; FFMQ). Residuals terms are not depicted. Loadings on indicator-specific method factor are not depicted and restrained to 1. λ = time invariant state factor loading. MSMQ = Multidimensional State Mindfulness Questionnaire; FFMQ = Five-Facet Mindfulness Questionnaire; AWA = acting with awareness, NJA = nonjudgmental acceptance, PMA = present-moment attention; NJ = nonjudge; NR = nonreact; DES = describe; OBS = observe; IS = indicator specific method factor.

In preparation for testing H2a, H2b, and H2c, we first fit a second-order latent growth model to state mindfulness (see Fig. 2 lower part). The results of this model with strong measurement invariance (CFI = 1.00; RMSEA = 0.004, 90% CI [0.00; 0.024]) replicated the results of the multilevel model: State mindfulness significantly increased over the training course (*M*_slope_ = 0.03, 95% CI [0.01; 0.06]), with different change rates between participants (*Var*_slope_ = 0.01, 95% CI [0.004; 0.01]; see further details in Supplementary Table S5).

In the next step, we calculated separate second-order latent growth model to examine trait change during the training (see Fig. 2 upper part, exemplarily for trait mindfulness; and Supplementary Table S5). Model fit with strong measurement invariance for trait mindfulness was good (CFI = 0.99; RMSEA = 0.04, 90% CI [0.02; 0.05]). Results indicate a significant increase in trait mindfulness over the timepoints (*M*_slope_= 0.14, 95% CI [0.10; 0.18]) at different change rates (*Var*_slope_ = 0.02, 95% CI [0.003; 0.04]). Similarly, trait self-compassion significantly increased throughout training participation (*M*_slope_ = 0.15, 95% CI [0.09; 0.20]; *Var*_slope_ = 0.03, 95% CI [0.002; 0.06]). However, we could only establish partial strong measurement invariance^74^ for self-compassion with the subscale Isolation being allowed to vary over time. The subscales suggest considerable heterogeneity and hence we caution with regards to model interpretation. Last, results of the single latent growth model for perceived stress (strong measurement invariance; CFI = 1.00; RMSEA = 0.00, 90% CI [0.00; 0.04]) suggest that overall means significantly decreased over the timepoints at differing rates (*M*_slope_ = − 0.14, 95% CI [− 0.19; −0.09]; *Var*_slope_ = 0.03, 95% CI [0.05; 0.07]). Detailed results on trait change during the training can be found in Supplementary Table S5 and in^70^.

### Correlated changes of state mindfulness and trait mindfulness, trait self-compassion, and trait perceived stress

With regards to the combined second-order latent growth models for state and trait mindfulness (see Fig. 2), model fit estimates suggested acceptable fit (CFI = 0.94; RMSEA = 0.04, 90% CI [0.04; 0.05]). Contrary to H2a, results indicate that increases in state mindfulness did not predict the increases in trait mindfulness (*b* = 0.04, 95% CI [− 0.12; 0.22]). Age did not moderate the state-trait relationship (*b* = − 0.03, 95% CI [− 0.52; 0.41]; see Supplementary Table S6). The model fit for the combined second-order latent growth model for state mindfulness and trait self-compassion was excellent (CFI = 0.99; RMSEA = 0.02, 90% CI [0.00; 0.024]). However, contrary to our expectations outlined in H2b, changes in state mindfulness did not predict the increases in trait self-compassion (*b* = 0.13, 95% CI [− 0.10; 0.38]). The correlated change between state mindfulness and trait self-compassion did not differ between younger or older adults (*b* = 0.17, 95% CI [− 0.30; 0.68]; see Supplementary Table S6). And last, contrary to H2c, the results of the combined second-order latent growth model with excellent fit (CFI = 0.98; RMSEA = 0.03, 90% CI [0.02; 0.03]) suggest that changes in state mindfulness did not predict decreases in perceived stress either (*b* = − 0.11, 95% CI [− 0.34; 0.12]). Again, the association in changes of state and trait was not moderated by age (*b* = − 0.07, 95% CI [− 0.55; 0.49]; see Supplementary Table S6).

## Discussion

This longitudinal study provides novel insights into the complex relationship between state trajectories of mindfulness with trait indicators of mindfulness, self-compassion, and perceived stress. The current findings extend previous research and theory by innovatively examining these state-trait associations in an 8-week Socioemotional Competence Training (SECT). More specifically, we replicated the previous finding that a psychological intervention without a specific mindfulness label led to increases in trait mindfulness (cf^49^. Additionally, the results of the current longitudinal study suggest that participation in the SECT led to significant improvements in state mindfulness, trait self-compassion, and trait perceived stress (cf^70^. Surprisingly, and contrary to our expectations, changes in state mindfulness did not predict changes in any of the traits. At a first glance, these findings contradict previous theoretical assumptions and former empirical findings from explicit MBIs^15,24,37,46,48^. However, our results may simply suggest a more nuanced state-trait relationship bearing important implications for both MBIs and other psychological interventions.

This study conveys a critical insight: Increases in state mindfulness alone appear insufficient to drive changes in trait mindfulness and other trait outcomes when the intervention is not explicitly mindfulness-based. Although trait mindfulness increased in the current study, this occurred without the significant state-trait association previously observed in traditional MBIs^15,46,48^. This pattern aligns with a subset of previous findings from personality intervention and psychotherapy research where no significant state–trait associations were observed (e.g^42,44,75^). Other studies, however, have reported significant associations^43,44^, suggesting that state–trait links may depend on moderating or mediation conditions. For example, Olaru et al.^43^ found that state–trait associations were stronger when participant commitment, task enjoyment, and task fulfillment were higher as well as when participants believed more strongly that personality traits are malleable. Zilcha-Mano^42^ highlighted that inconsistent result patterns may reflect differential roles of trait-like and state-like components during therapy. This framework underscores how within-treatment state-like processes can serve as mechanisms that drive longer-term trait change if taken into account that trajectories may not always have the same direction. For example, lower baseline (trait-like) treatment expectancy may hinder therapeutic success, whereas session-to-session (state-like) increases in expectancy may predict greater success.

The current findings contribute to the ongoing debate about common and specific change mechanisms across different intervention types^36,42,76^. Traditional MBIs seem to contain specific elements beyond mindfulness exercises that strengthen state-trait associations found previously. Although the SECT included mindfulness practices (e.g., attention to the body or breath), these were not embedded in a mindfulness psychoeducational context and required substantially less daily practice time than traditional MBIs (10–20 min vs. 45 min daily in traditional MBIs^49^). According to theoretical assumptions of the WTT, the repeated experience of more mindfulness in daily life should result in trait change over time^15^. However, the reduced dose in the SECT may not have generated sufficient state mindfulness experiences to reach a threshold for meaningful state-trait association. Previous research shows that the outcome is reciprocally linked with practice time^77^, and that a perceived positive outcome may be a better predictor of longer practice times than vice versa^78^. Hence, especially without specific mindfulness framing of the exercises, participants may have prioritized other SECT exercises—potentially because they were more obviously aligned with the SECT goals or because they were perceived as more beneficial. This could also explain the trait improvements in downstream outcomes, that is self-compassion and perceived stress. In addition to practice time, trainer characteristics likely influence state-trait associations. SECT teachers did not have to follow a mindfulness practice of their own, as often required from MBSR teachers^5^. Yet, trainers’ embodiment of mindfulness principles may facilitate participants’ recognition of state-trait connections through explicit instruction and implicit modeling.

Trait improvements in the SECT may have occurred through alternative pathways, such as increased emotional stability, or engagement in broader reflective or other self-regulatory processes^24,44^. This aligns with perspectives on general, pan-theoretical change mechanism in psychotherapy, which suggest that increased differentiation between external triggers and affective reactions represents a common therapeutic mechanism^79^. Mindfulness can be one means to create this emotional space between a trigger and the response to it^28,29^ but may exert these effects only through explicit instruction in MBI contexts. Hence, we argue that state mindfulness changes may be necessary but not sufficient for trait mindfulness development, particularly in the absence of mindfulness-specific elements that characterize traditional MBIs. A similar mechanism may also hold for the related construct of self-compassion, as previous research suggests (e.g^67^). Conversely, reductions in perceived stress can also occur through alternative pathways (e.g., cognitive restructuring in CBT), underscoring the need for future research to disentangle the relative contribution of different change mechanisms across interventions.

Another intriguing thought comes from personality change research, which suggests that explicit awareness and reflection of behavioral (state) changes and their attribution to aggregated self-perceptions may be central to translating momentary experiences into lasting trait-level change^41,44^. Mindfulness may facilitate the awareness part, but conscious reflection may need more instruction or explicit framing. Given that the SECT included mindfulness exercises but did not provide a comprehensive contemplative framework, participants may have attributed state mindfulness fluctuations to momentary and contextual influences rather than enduring trait change. Additionally, state and trait changes may not occur for all individuals equally: Some participants may show behavioral (state) changes without self-concept integration while others may exhibit self-concept changes without obvious behavioral adaptation. How to measure and overcome the informational loss in state-trait translations during an intervention—and whether it affects the size, stability, or the duration of intervention effects—remains an intriguing question for future research.

Our results may also point toward expectancy effects. Participant expectations and intentions shape both subjective experiences and objective outcomes^42,80–82^, potentially explaining why state-trait relationships differ between intervention types despite similar directional improvements. These expectations are, among others, created by contextual framing of exercises and program structure (see above^15,80^). While participants in MBIs likely anticipate improvements in mindfulness and reductions in stress—for example when participating in MBSR—the SECT addressed individuals seeking to become more relaxed in face of daily stress and to handle challenging social situations more calmly. Participants in MBIs may experience greater trait changes specifically because they anticipate mindfulness benefits, receive explicit framing about mindfulness mechanisms, and are instructed to attribute their state experiences to broader trait changes (cf^83^.. In contrast, the mindfulness exercises incorporated in the SECT were presented as (attention and emotion) regulation techniques rather than mindfulness training. This expectancy divergence is reflected in the effect sizes, which are medium in size but still considerably smaller (*d* = 0.62 for mindfulness, *d* = − 0.51 for perceived stress) than those reported in traditional MBIs (up to *d* = 1.14 for trait mindfulness and *d*= − 0.64 to − 1.04 for perceived stress^46,48^).

Taken together, our results suggest interesting practical implications that can be considered in future intervention design and evaluation. The results highlight that interventions may benefit from making the goals and rationale of training explicit—clearly explaining why certain exercises are included and reinforcing participants’ belief in their efficacy. This is in line with previous suggestions to use patients’ expectancy as an active ingredient^37^ (see^80^ for an illustrative example). Moreover, recent advances in the use of artificial intelligence for adaptive intervention design offer promising avenues to identify which components, or which types of training, are most effective for different individuals^84–86^. Such precision-oriented approaches may help maximize the translation of state-level experiences into lasting trait change. This would also help to disentangle whether state- and trait-level changes unfold independently (or in different directions, cf^.42^), or whether one is more influential than the other for long-term maintenance. For example, state changes may reflect short-term fluctuations, but may also depict the initial integration of new behaviors into everyday life.

### Limitations

Our findings should be considered in light of important limitations. First, measuring state mindfulness retrospectively over one week has been criticized as lacking the fine-grained resolution necessary for studying momentary fluctuations^12^, potentially representing trait-like rather than state characteristics (cf^15^. The retrospective aggregation of mindfulness experience over the past week might obscure day-to-day fluctuations and thereby limit variance in our measures. Previous findings show that ecological momentary assessment detected intervention effects of an MBSR program on anxiety and depression that self-report trait measures apparently failed to capture^87^. This divergence highlights limitations of trait—and potentially weekly—self-report assessments in capturing dynamic psychological processes, potentially depicting biased snapshots of actual functioning.

Second, reliance on self-report measures with their typical short-comings as well as using different instruments for state (Multidimensional State Mindfulness Questionnaire^12^) versus trait mindfulness (Five-Facet Mindfulness Questionnaire^6^), potentially measuring distinct constructs, may limit our ability to capture true state-trait associations. Yet, our study was the first to use two validated measures in their intended purpose, whereas previous research relied on adapted trait measures or ad-hoc designed questions (e.g^15,46,48,88^). Future research could employ more fine-grained measures to better understand state-trait relationships in mindfulness interventions, for example, by using validated momentary assessments in ecological momentary assessment periods during the intervention or neurobiological indicators of mindfulness. However, repeatedly measuring momentary state mindfulness may be an intervention in itself, reminding participants of their intention to be non-judgmentally aware of the present-moment^81^.

Third, we did not include measures of weekly changes in self-compassion or perceived stress. It would be interesting to examine reciprocal associations of these trajectories in future research, in both explicit (compassion or stress-reduction) interventions and broad psychological interventions such as the SECT, in order to test whether the current result patterns generally replicate.

Last, several sample-related limitations should be noted. Due to the design of an in-person psychological intervention, although spanning a large age range, our sample was mostly female, highly educated, and with a comparatively high socio-economic status. Future research is needed to determine whether the current results extend to more heterogenous, minority, or clinical populations, ideally with additional measures, such as observer reports or (neuro-)biological markers of state and trait variables. Moreover, despite intensive recruitment efforts, we were not able to reach the sample size indicated by our a priori power analysis (*N* = 220) for detecting medium-to-large effects. As a result, the null findings regarding state–trait associations should be interpreted with caution, as reduced statistical power may be an explanation for the current result patterns.

The current findings suggest that mindfulness-based practices can be integrated into a Socioemotional Competence Training, but they may not exert the same effects as in explicit MBIs due to person, context, and process specific and common change mechanisms. Trait changes occurred even without a strong state-trait association, which suggests a complex relationship between state and trait variables in this study, and potentially in psychological interventions using mindfulness techniques without explicitly labeling them more generally. Replication of these findings with a larger and more heterogeneous sample, in other psychological interventions without explicit mindfulness labeling, and potentially different variables may make the assumptions drawn here more reliable.

## Method

### Transparency and openness

Hypotheses were preregistered on the Open Science Framework (OSF; see minor deviations from the preregistration in Supplementary Table S7). The anonymized dataset, analysis code, and code book with an overview of all variables of the project are also stored on the OSF. Data from the same dataset but with distinct research questions have been previously used in^44,70^. The study was approved by the Ethics Committee of the Faculty of Behavioural and Cultural Studies at Heidelberg University, Germany (Agu 2022 4/1) and aligns with the principles of the Declaration of Helsinki. We obtained informed consent from all study participants prior to participation.

### Procedure

Participants were recruited on- and offline (e.g., through social media advertising, flyers, or talks at various institutions). We provided a link through which interested people reached the online survey platform SosciSurvey^89^. After giving informed consent approximately 1,150 people were screened for study eligibility criteria, that included (1) belonging to one of the two age groups of younger (18–42 years) or older (50–78 years)^2^ adults for other research questions, (2) appropriate access to study material and questionnaires, and (3) no participation in another training, or psychotherapy/psychological counseling. Moreover, respondents who (4) exceeded clinical cut-off values for depression and/or generalized anxiety were excluded^90,91^. In total, of the 560 participants who were eligible for the training, 203 enrolled in it, and 166 ultimately finished the training.

Following successful screening, participants were informed on the study purpose and requirements, data collection and privacy, as well as training cost and reimbursement. Informed consent was collected a second time at this point. To ensure higher participant commitment and adherence to the training, participants paid EUR 80 (or EUR 50 at a reduced rate) for participation. After their participation, they were reimbursed with up EUR 110 plus half of the training fee depending on their adherence to the training sessions and study requirements. While filling out the baseline questionnaires, participants were randomly allocated to either a training group or a waitlist control group. Because this study only assessed state mindfulness during active training participation, we did not include analyses on group comparisons. Additional information on recruitment, enrollment, screening, random group allocation, and drop-out of participants is detailed in^27,70^.

### Socio-emotional competence training

Participants took part in an 8-week Socio-Emotional Competence Training (SECT) with weekly 2-hours sessions. Group size ranged from five to twelve participants. Each group was led by two graduate students who were trained by the principal investigators, CAR and CW. In total, 27 trainers conducted one or more of the 22 training groups that took place between January 2023 and June 2023.

The training consisted of two parts. The first 4 training weeks concentrated on the development of emotional competences. Participants learned about stress synthesis, resilience, as well as attention and emotion regulation through psychoeducation, self-reflection, and various practical exercises (e.g., body awareness, meditation, noticing of stressful emotions). Based on this knowledge and while continuing the practical exercises, the next 4 weeks concentrated on strengthening social competences. Participants were educated on systemic perspectives on social dynamics, and practiced new behavior in fictious videotaped role plays with the trainers. In-between training sessions, participants were asked to continue learning and practicing in their daily lives by themselves and with a training buddy, via self-reflection, guided audio exercises, and behavioral tasks (e.g., practicing new and/or challenging social behavior). For more detailed information on the training structure and content see^70^.

### Sample size

In consideration of feasibility issues with conducting an RCT with a training and a waitlist control group, as well as different research questions demanding two age groups (*N* = 110 young adults, 18–35 years, *N* = 110 older adults, 55–80 years), power analyses (1-β = 0.80, α = 0.05) suggested that 220 participants would be needed to find medium and large effect sizes. As mentioned, 203 participants enrolled in the training, and 166 participants finished at least four out of eight training sessions.

### Measures

All trait questionnaires were administered before (T1), during (T2), and after the training (T3). Data from the follow-up timepoints, 3 and 12 months (T4 and T5, respectively) after the end of the training, were not included in the current analyses. Data on weekly mindfulness were collected 4 to 6 days after each of the eight training modules.

 State Mindfulness was measured with the Multidimensional State Mindfulness Questionnaire (MSMQ^12^). The questionnaire measures three facets of state mindfulness: Acting with Awareness, Nonjudgmental Acceptance, and Present-Moment Attention. The 9 items were answered on a 6-point Likert scale ranging from *does not apply at all* to *applies strongly* and showed good reliability in the current sample (ω = 0.74 − 0.82). State mindfulness was assessed weekly, approximately 5 days after each training module. We chose this timing to minimize short-term reactivity to single sessions and instead capture participants’ mindfulness experiences across the training week. This approach follows theoretical work conceptualizing traits as relatively stable self-concepts that emerge from the repeated enactment of states^15,40,41^. Weekly measures therefore reflect proximal behaviors and experiences, in contrast to trait measures, which assess broader and more enduring self-concepts.

 Trait Mindfulnesswas assessed with the Five Facet Mindfulness Questionnaire (FFMQ^92^), measuring the five facets Describe, Observe, Act with Awareness, Nonjudge (8 items each), and Nonreact (7 items) with a total of 39 items on a 5-point Likert scale ranging from *never or very rarely true* to *very often or always true*. Reliability in the current sample was good (ω = 0.90 − 0.92).

 Self-Compassion was assessed with the short version of the Self-Compassion Scale (SCS^93^). The scale consists of 12 items that measure the six dimensions of self-compassion (two items per dimension; positive: Mindfulness, Self-Kindness, Common Humanity; negative: Overidentification, Self-Criticism, Isolation) on a 5-point Likert scale ranging from *almost never* to *almost always*. McDonald’s ω indicated good reliability of the scale in the current sample (ω = 0.87 − 0.91).

 Perceived Stress was assessed with the 10-item Perceived Stress Scale (PSS^94^) on a 5-point Likert scale ranging from *never* to *very often*. The scale measures two dimensions of perceived stress, that is, the perceived helplessness (6 items) and self-efficacy (4 items, reverse coded) to deal with stressors over the past two weeks and showed good reliability in this sample (ω = 0.84 − 0.91).

### Data analyses

Data were analyzed with R, version 4.3.1^95^; see Supplemental Material for used R packages) and MPlus, version 8.6^96^. In sum, 10.8% of data were missing (1,379 out of 11,403 data points). Moreover, we detected 16 outliers (out of 237,319 data points) and winsorized them to *M* ± 3 *SD* for further analyses.

To test for the increases in state mindfulness, we calculated multilevel analyses with time as a Level 1 random within-person predictor (coded from 0 to 7). We included age group (0 = younger, 1 = older adults) as a grand-mean centered Level 2 predictor, given that other research questions required these two age groups (e.g., see^44^. By including a cross-level interaction between Time x Age Group we examined whether state mindfulness developed differently between older (= 0) and younger adults (= 1).

To test Hypotheses 2a, 2b, and 2c, whether state mindfulness predicted trait change, we first fitted second-order latent growth models to state mindfulness and each trait variable separately (see minor deviation in analysis strategy from preregistered analyses in Supplementary Table S7). These models should replicate findings from the multilevel analyses conducted to test H1 and for the trait increases as found in^70^. In a second step, we regressed the latent trait slope on the latent state slope (see Fig. 2; exemplarily for state and trait mindfulness). For the trait slope, loadings were set to 0, 4, and 8 to reflect the approximate 4-week intervals between the three timepoints. Loadings for the state slope were set to 1 through 8 across the eight weekly assessments. We slightly changed the time coding in order to depict the time delay in trait and state questionnaires. Results with a coding from 0 to 7 did not change results. To examine whether the state-trait relationship differed with age, we extended the latter models with age group as a moderator in the relationship and regressed the respective trait slope on the interaction of state slope with age group.

Latent growth analyses were estimated with Bayes estimator with 20.000 iterations (10.000 iterations in the extended age model), because estimation with MLR for state mindfulness and combined models did not converge, potentially because of high complexity of these models and a comparatively small sample size. Default, non-informative priors were used with two chains (Markov Chain Monte Carlo with Gibbs sampler), in which the first half is considered burn-in^97^. Convergence was determined by evaluating trace plots and examining the Potential Scale Reduction Factor (PSRF), with values > 1.1 indicating convergence^97,98^. Convergence was double-checked with a higher number of iterations^97^. Estimates and PSRF with higher iterations did not essentially differ. The analysis provides point estimates and 95% credibility intervals (CI) of the posterior distribution. Effects are considered significant if the CI does not include zero.

In all models, mindfulness, self-compassion, and perceived stress were modeled as latent variables. Items were parceled according to the respective subscales of the three trait measures (e.g., Describe, Observe, Nonjudge, Nonreact, and Act with Awareness for the FFMQ), which then loaded on the respective latent trait. All models included indicator-specific method factors, uncorrelated with all other variables, which offer more reliable estimations than correlated residuals^99^. Based on theoretical and data-driven (i.e., sensitivity analyses) considerations, the facets/subscales Nonreact (FFMQ), Self-Kindness (SCS), and Self-Efficacy (PSS) were chosen as reference factors, respectively^99^. Strong and partial strong measurement invariance was separately determined before calculating all final models^74,99^ (see Supplementary Table S8). Overall, model fit for all models was evaluated using the standard cutoff criteria in psychological research^100^, where comparative fit index (CFI) values ≥ 0.90 and root-mean-square error of approximation (RMSEA) values ≤ 0.08 indicate an acceptable fit.

## Supplementary Information

Below is the link to the electronic supplementary material.

Supplementary Material 1

## Data Availability

The preregistration, data, and script are openly available on the Open Science Framework (OSF) at [https://osf.io/jnc9e/](https:/osf.io/jnc9e/) and [https://osf.io/q3wft/](https:/osf.io/q3wft/).

## Footnotes

1 One participant did not complete the main questionnaires but all weekly assessments and was thus retained in the analysis.

2 Because of severe recruitment challenges, we included six middle-aged adults. Originally preregistered age ranges were 18-35 years and 55-80 years.
