# Supplementary material for: Intervention-induced changes in state mindfulness do not predict trait changes in mindfulness, self-compassion, or perceived stress
Source: Sci Rep. 2025 Nov 21;15:41174. doi: 10.1038/s41598-025-27697-0 (PMC12639022; doi:10.1038/s41598-025-27697-0)
Supplement: Supplementary file 1 — Supplementary Material 1 [file 41598_2025_27697_MOESM1_ESM.pdf]

Supplementary Material

Intervention-induced changes in state mindfulness do not predict trait changes in mindfulness, self-compassion, or perceived stress

Borgdorf, K. S. A., Küchler, G., Wrzus, C., & Aguilar-Raab, C.

Table S1
Sociodemographic Information

Variable	Overall (N = 165)	Younger adults (n = 81)	Older adults (n = 84)
**Age**			
Mean (SD)	46.26 (18.70)	28.33 (4.92)	63.55 (7.20)
Range	19-78	19-42	50-78
**Gender *n*(%)**			
Female	124 (75.15)	61 (75.31)	63 (75.00)
Male	40 (24.24)	19 (23.46)	21 (25.00)
Non-Binary	1 (0.61)	1 (1.23)	0 (0.00)
**Education *n*(%)**[a]			
Up to middle school degree	14 (8.48)	2 (2.47)	12 (14.29)
High school degree	29 (16.97)	21 (25.93)	8 (9.52)
College degree (UAS)	5 (3.03)	1 (1.23)	4 (4.76)
University degree	104 (63.03)	53 (65.43)	51 (60.71)
Other	9 (5.45)	4 (4.94)	5 (5.95)
**Job *n*(%)**[a]			
Student	45 (27.27)	45 (55.56)	0 (0.00)
Employee	65 (39.39)	30 (37.04)	35 (41.67)
Self-Employed	4 (2.42)	0 (0.00)	4 (4.76)
Pensioner	36 (21.82)	0 (0.00)	36 (42.86)
Other	11 (6.67)	6 (7.41)	5 (5.95)
**Civil Status *n*(%)**[a]			
Married	48 (29.09)	9 (11.11)	39 (46.43)
In a relationship	41 (24.85)	36 (44.44)	5 (5.95)
Single	47 (28.48)	35 (43.21)	12 (14.29)
Divorced	19 (11.52)	1 (1.23)	18 (21.43)
Widowed	6 (3.64)	0 (0.00)	6 (7.14)
**Kids *n*(%)**[a]			
None	97 (58.79)	76 (93.83)	21 (25.00)
1	17 (10.30)	3 (3.70)	14 (16.67)
2	35 (21.21)	1 (1.23)	34 (40.48)
3	9 (5.45)	1 (1.23)	8 (9.52)
4	3 (1.82)	0 (0.00)	3 (3.54)
**Net Household Income in € *n*(%)**[a]			
< 1.000	41 (24.85)	33 (40.74)	8 (9.82)
1.000 - 2.000	31 (18.79)	14 (17.28)	17 (20.24)
2.000 - 3.000	42 (25.45)	18 (22.22)	24 (28.57)
3.000 - 5.000	29 (17.58)	10 (12.35)	19 (22.62)
5.000 - 7.500	6 (3.64)	0 (0.000)	6 (7.14)
7.500 - 10.000	2 (1.21)	0 (0.00)	2 (2.38)
> 10.000	1 (0.61)	0 (0.00)	1 (1.19)
Not indicated	9 (5.45)	6 (7.41)	3 (3.57)
**Meditation Experience *n*(%)**[c]			
yes, of these	81 (50.31)	39 (48.15)	42 (52.50)
< 1 year, *n*(%)	39 (48.15)	18 (46.15)	21 (50.00)
> 1 year, *n*(%)	40 (49.38)	21 (53.85)	19 (45.24)
Not at all - once a month	49 (60.49)	22 (56.41)	27 (64.29)
Several times a month - daily	32 (39.51)	17 (43.89)	15 (35.71)

Note. Adpated from Borgdorf et al. (2025). UAS = University of Applied Sciences. [a]n = 4 missings in overall and older age group. [b]Categories collapsed into df+1 categories for analysis purposes (see OSF script). [c]n = 2 missings in overall and older age group.

Table S2

Dropout Analyses

Variables *M(SD)*	Completers (N = 165)[a]	Drop-out (N = 38)[a]	Welch's $t(df)$ or $\chi^2(df)$[b]	p
Mindfulness	3.26 (0.44)	3.28 (0.44)	0.27 (43.83)	.790
Self-Compassion	2.93 (0.64)	2.87 (0.51)	−0.55 (49.75)	.586
Perceived Stress	2.94 (0.58)	3.11 (0.56)	1.54 (43.24)	.132
Age	46.26 (18.70)	47.81 (18.60)	0.46 (53.58)	.649
Gender (*n*)			1.45 (2)	.484
Female	124	24		
Male	40	12		
Non-binary	1	0		
Meditation experience; *n*(%)				
% yes, of these	50.3%	55.6%	0.15	.700
< 1 year	20 (25.0)	6 (30.0)	2.10	.954
> 1 year	61 (75.3)	14 (70.0)		
Not at all - once a month	49 (60.5)	14 (70.0)	4.31	.635
Several times a month - daily	32 (39.5)	6 (30.0)		

Note. 36.3% of the eligible participants (N = 203) eventually enrolled in the study. Of these, 18.7% (n = 38) decided not to start (n = 20) or to end participation prematurely (i.e., did only complete ≤ 4 training sessions; n = 18). These are considered a drop-out. [a]Data is available from $n_{\text{Completers}}$ = 158-165; $n_{\text{Drop-out}}$ = 30-37 at T1. [b]Mean difference at T1 between completers and drop-out. Adapted with friendly permission from Borgdorf et al. (2025).

Table S3

Cohort Comparison at T1

Variables *M* (*SD*)	January $N = 48$	April $N = 52^a$	June $N = 65^a$	*F (df)* or $\chi^2(df)$	*p*
Age					
Mean (SD)	47.33 (19.02)	41.88 (18.50)	48.97 (18.27)	0.22 (1)	.639
Range	20-75	20-78	19-78		
Gender *n*(%)					
Female	36 (75.0)	37 (71.15)	51 (78.46)		
Male	11 (22.92)	15 (28.85)	14 (21.54)	3.33 (4)	.504
Non-Binary	1 (2.78)	0 (0.00)	0 (0.00)		
Mindfulness	3.31 (0.41)	3.23 (0.48)	3.25 (0.42)	0.39 (1)	.534
Self-Compassion	2.96 (0.59)	2.95 (0.73)	2.89 (0.60)	0.35 (1)	.553
Perceived Stress	2.93 (0.54)	2.97 (0.61)	2.92 (0.59)	0.01 (1)	.918
Meditation experience *n*(%)					
yes, of these	25 (52.08)	26 (50.00)	30 (49.18)	0.09 (2)	.954
< 1 year	11[b] (44.00)	13 (50.00)	15 (50.0)	20.74 (14)	.108
> 1 year	12[b] (48.00)	13 (50.00)	15 (50.00)		
Not at all - once a month	15 (60.00)	13 (50.00)	21 (70.00)	8.15 (12)	.773
Several times a month - daily	10 (40.00)	13 (50.00)	9 (30.00)		

Note. [a]Data is available from $n_{\text{April}} = 51$ and $n_{\text{June}} = 61$. [b]$n = 2$ missing values. Adapted from Borgdorf et al. (2025).

Table S4

Descriptive Statistics, Reliabilities, and Bivariate Correlations

	Variables	*N*	*M* (*SD*)	*d* [a]	ω	1	2	3	4	5	6	7	8	9	10	11	12	13	14	15	16
	**MSMQ**																				
1	Week 1	155	3.82 (0.76)		.78																
2	Week 2	155	3.90 (0.70)	0.15	.74	.60															
3	Week 3	151	3.88 (0.67)	0.09	.77	.59	.66														
4	Week 4	148	3.95 (0.72)	0.19	.77	.48	.57	.62													
5	Week 5	149	4.08 (0.67)	0.29	.75	.49	.63	.56	.65												
6	Week 6	144	4.17 (0.72)	0.43	.82	.46	.52	.52	.63	.61											
7	Week 7	142	4.15 (0.71)	0.43	.76	.45	.58	.63	.63	.62	.62										
8	Week 8	134	4.15 (0.70)	0.38	.78	.45	.55	.59	.60	.61	.59	.75									
	**FFMQ**																				
9	T1	156	3.24 (0.45)		.89	.57	.57	.53	.48	.54	.45	.49	.42								
10	T2	149	3.34 (0.48)	0.30	.92	.61	.57	.61	.60	.64	.51	.55	.48	.82							
11	T3	141	3.46 (0.45)	0.62	.91	.55	.50	.57	.58	.57	.57	.50	.53	.72	.83						
	**SCS**																				
12	T1	156	2.98 (0.65)		.87	.46	.47	.43	.42	.48	.39	.47	.36	.70	.61	.56					
13	T2	148	3.08 (0.66)	0.23	.89	.50	.50	.49	.51	.55	.45	.52	.43	.62	.68	.67	.80				
14	T3	141	3.26 (0.69)	0.51	.91	.42	.39	.44	.39	.44	.42	.44	.43	.50	.57	.69	.73	.83			
	**PSS**																				
15	T1	156	2.92 (0.60)		.87	-.23	-.37	-.28	-.28	-.40	-.29	-.27	-.17	-.42	-.40	-.37	-.52	-.51	-.41		
16	T2	149	2.79 (0.65)	-0.26	.90	-.37	-.44	-.40	-.41	-.39	-.42	-.39	-.29	-.41	-.50	-.48	-.41	-.59	-.54	.59	
17	T3	140	2.62 (0.65)	-0.51	.91	-.29	-.32	-.36	-.26	-.30	-.31	-.34	-.32	-.35	-.36	-.50	-.42	-.50	-.63	.60	.66

Note. MSMQ = Multidimensional State Mindfulness Questionnaire; FFMQ = Five Facet Mindfulness Questionnaire, SCS = Self-Compassion Scale Short-Form; PSS = Perceived Stress Scale. All $r \geq |.23|$ are significant at $p < .05$. [a] Paired Cohen's *d* for repeated measures between pre and last post measure (Week 1 and Week 8/ T1 and T3), respectively.

Table S5

Results for Separate Second-Order Latent Growth Model and State-Trait Associations

Model	Estimate	95% CI	Posterior SD	CFI	RMSEA [90% CI]
**State Mindfulness**				1.00	0.00 [0.00, 0.02]
Mean Intercept	3.76	[3.61, 3.90]	0.07		
Mean Slope	0.03	[0.01, 0.06]	0.01		
Variance Intercept	0.57	[0.41, 0.77]	0.08		
Variance Slope	0.01	[0.00, 0.01]	0.00		
Std. Correlation	−0.32	[−0.54, −0.02]	0.15		
**Trait Mindfulness**				0.99	0.04 [0.02, 0.05]
Mean Intercept	2.78	[2.68, 2.88]	0.05		
Mean Slope	0.14	[0.10, 0.18]	0.02		
Variance Intercept	0.32	[0.24, 0.43]	0.05		
Variance Slope	0.02	[0.00, 0.04]	0.00		
Std. Correlation	−0.32	[−0.54, −0.02]	0.15		
**State → Trait Mindfulness**				0.94	0.04 [0.04; 0.05]
Trait Slope on State Slope	0.04	[−0.12, 0.22]	0.09		
Std. Correlation Intercepts	0.40	[0.33, 0.56]	0.09		
**Trait Self-Compassion**				0.97	0.05 [0.04, 0.06]
Mean Intercept	2.96	[2.84, 3.07]	0.06		
Mean Slope	0.15	[0.09, 0.20]	0.03		
Variance Intercept	0.39	[0.28, 0.52]	0.06		
Variance Slope	0.03	[0.00, 0.06]	0.02		
Std. Correlation	−0.06	[−0.41, 0.61]	0.26		
**State → Trait Self-Compassion**				0.99	0.02 [0.00; 0.02]
Trait Slope on State Slope	0.13	[−0.10, 0.38]	0.12		
Std. Correlation Intercepts	0.41	[−0.61, −0.11]	0.13		

Table S5 (contd.)

Results for Separate Second-Order Latent Growth Model and State-Trait Associations

Model	Estimate	95% CI	Posterior SD	CFI	RMSEA [90% CI]
**Trait Perceived Stress**				1.00	0.00 [0.00, 0.04]
Mean Intercept	2.74	[2.65, 2.83]	0.05		
Mean Slope	−0.14	[−0.19, −0.09]	0.03		
Variance Intercept	0.24	[0.16, 0.35]	0.05		
Variance Slope	0.03	[0.01, 0.07]	0.02		
Std. Correlation	−0.06	[−0.43, 0.64]	0.27		
**State → Trait Perceived Stress**				0.98	0.03 [0.02; 0.03]
Trait Slope on State Slope	−0.11	[−0.34, 0.12]	0.12		
Std. Correlation Intercepts	−0.33	[−0.51, −0.13]	0.10		

Note. Results of the separate second-order latent growth model and change in state mindfulness predicting trait change, respectively. Models were run with Bayes estimator and 10,000 iterations.

Table S6

State-Trait Associations Moderated by Age Group (Extended Model)

Model	Estimate	95% Credibility Interval	Posterior SD
**Trait Mindfulness**			
Trait Change on State Change	0.05	[−0.19, 0.29]	0.12
Trait Change on State Change x Age Group	−0.03	[−0.52, 0.41]	0.24
**Trait Self-Compassion**			
Trait Change on State Change	0.10	[−0.18, 0.36]	0.14
Trait Change on State Change x Age Group	0.17	[−0.30, 0.68]	0.26
**Trait Perceived Stress**			
Trait Change on State Change	−0.08	[−0.38, 0.16]	0.14
Trait Change on State Change x Age Group	−0.07	[−0.55, 0.49]	0.25

Note. Results of the latent growth models with change in state mindfulness predicting trait change, respectively, including age group as moderator of this relationship. Models were run with Bayes estimator and 10,000 iterations. Age group was grand mean centered.

Table S7

Deviations from the Preregistration

Preregistration	Deviation	Reasoning
Hypothesis 2c (H2c): More pronounced changes in state mindfulness are associated with stronger changes in trait **stress regulation.**	Hypothesis 2c (H2c): More pronounced changes in state mindfulness are associated with stronger changes in trait **perceived stress**.	We changed the wording slightly to align more closely with the measurement instrument (i.e., Perceived Stress Scale) preregistered and used.
"Trait mindfulness will be measured using the 39-item Five Facet Mindfulness Questionnaire […] and two subscales of the Comprehensive Inventory of Mindfulness Experiences […] Mean score and facet level analyses."	We did not include the CHIME subscales.<br><br>We did not conduct analyses on the subscale level.	We did not include CHIME subscales due to mixed results in previous project parts (see blinded and blinded).<br><br>We did not include subscales, because of bad model fit—presumably because of the comparatively small sample size/power reasons.
"For H2a to H2c: If the data quality is sufficient, multilevel structural equation models are specified that link changes in weekly experiences (i.e., slopes) with trait changes (i.e., latent neighbor change models)."	We do not report results of latent neighbor change models. Instead, we report results of latent growth models.	The combination of latent growth models (state) and latent neighbor models (trait) resulted in bad model fit. The combination of two second-order latent growth models was more suitable and resulted in good model fit. Moreover, model estimates for separate latent growth and latent neighbor models for the trait variables did not differ meaningfully.
"Control variables: Compliance with training tasks (4 items), experience with meditation (2 items)"	We do not report these analyses.	The control variables did not significantly moderate the relationship between state mindfulness and trait variables.
"We will also examine whether state changes in mindfulness are associated with stronger changes in related variables, i.e. empathy and perspective taking."	We did not conduct these analyses.	We wanted to keep the focus of the paper concise. Also, the SECT did not yield significant increases in empathy and perspective taking (see blinded).

Table S8

Comparison of Measurement Invariance in Separate Latent Change Models

Model	χ^2 (df)	BIC	CFI	RMSEA [90% CI]
**State Mindfulness**[a]				
Configural Invariance		8362.99	0.99	0.02 [0.00, 0.04]
Weak Invariance		8232.36	1.00	0.01 [0.00, 0.03]
Strong Invariance		8222.19	1.00	0.01 [0.00, 0.03]
**Trait Mindfulness**				
Configural Invariance	98.63 (69)	3112.79	0.98	0.05 [0.03, 0.07]
Weak Invariance	125.30 (85)	3058.41	0.98	0.05 [0.03, 0.07]
Strong Invariance	139.13 (93)	3031.39	0.97	0.06 [0.04, 0.07]
**Trait Self-Compassion**				
Configural Invariance	150.75 (107)	5275.51	0.98	0.05 [0.03, 0.07]
Partial[b] Weak Invariance	191.27 (125)	5224.13	0.97	0.06 [0.04, 0.07]
Partial[b] Strong Invariance	213.64 (133)	5205.66	0.96	0.06 [0.02, 0.05]
**Trait Perceived Stress**[a]				
Configural Invariance		1396.07	1.00	0.00 [0.00, 0.11]
Weak Invariance		1376.57	1.00	0.00 [0.00, 0.07]
Strong Invariance		1368.04	1.00	0.00 [0.00, 0.06]

Note. BIC = Bayesian Information Criterion; CFI = Comparative Fit Index; RMSEA = Root Mean Square Error of Approximation. For testing measurement invariance, we followed the guidelines of Chen (2007) for samples with N ≤ 300. [a]Bayes estimation with 10,000 iterations (because estimation with MLR did not converge). [b]Subfacet Isolation being allowed to vary over time.

R packages and References

Auguie, B., & Antonov, A. (2017). *gridExtra: Miscellaneous functions for "Grid" graphics* (R package version 2.3). https://CRAN.R-project.org/package=gridExtra

Bates, D., Mächler, M., Bolker, B., & Walker, S. (2015). Fitting Linear Mixed-Effects Models Using lme4. *Journal of Statistical Software*, *67*(1), 1-48. https://doi.org/10.18637/jss.v067.i01

Beaujean, A. A. (2012). *psychometric: Applied Psychometric Theory*. https://CRAN.R-project.org/package=psychometric

Ben-Shachar, M. S., Lüdecke, D., & Makowski, D. (2020). effectsize: Estimation of Effect Size Indices and Standardized Parameters. *Journal of Open Source Software, 5*(56), 2815, https://doi.org/10.21105/joss.02815

Borgdorf, K. S. A., Küchler, G., Wrzus, C., & Aguilar-Raab, C. (2025). Mindful and well: The effects of a socioemotional competence training (SECT) in a randomized controlled trial. *Journal of Counseling Psychology, 72*(4), 329–341. https://doi.org/10.1037/cou0000802

Brown, A. (2021). *careless: Procedures for Computing Indices of Careless Responding*. https://CRAN.R-project.org/package=careless

Chen, F. F. (2007). Sensitivity of goodness of fit indexes to lack of measurement invariance. *Structural Equation Modeling: A Multidisciplinary Journal, 14*(3), 464–504. https://doi.org/10.1080/10705510701301834

Hallquist MN, Wiley JF (2018). MplusAutomation: An R Package for Facilitating Large-Scale Latent Variable Analyses in Mplus. *Structural Equation Modeling*, 621–638. doi:10.1080/10705511.2017.1402334,

Harrell Jr, F. E. (2023). *Hmisc: Harrell Miscellaneous*. https://CRAN.R-project.org/package=Hmisc

Leifeld, P. (2013). texreg: Conversion of Statistical Model Output in R to LaTeX and HTML Tables. *Journal of Statistical Software*, 55(8), 1–24. https://doi.org/10.18637/jss.v055.i08.

McNamara, A., Ellis, S., & Waring, E. (2022). *skimr: Compact and flexible summaries of data*. https://CRAN.R-project.org/package=skimr

Kelley, K. (2017). *MBESS [computer software and manual]*. Accessible from http://cran.r-project.org.

Revelle, W. (2024). psych: *Procedures for psychological, psychometric, and personality research. R package version 2.3.6*. https://CRAN.R-project.org/package=psych

Rosseel, Y. (2012). lavaan: An R Package for Structural Equation Modeling. *Journal of Statistical Software, 48*(2), 1-36. https://doi.org/10.18637/jss.v048.i02

Signorell, A., et mult. al. (2023). *DescTools: Tools for descriptive statistics*. R package version 0.99.50. https://CRAN.R-project.org/package=DescTools

Torchiano M (2020). *effsize: Efficient Effect Size Computation*. https://CRAN.R-project.org/package=effsize

Wickham, H., Averick, M., Bryan, J., Chang, W., McGowan, L. D. A., François, R., Grolemund, G., Hayes, A., Henry, L., Hester, J., Kuhn, M., Pedersen, T. L., Miller, E., Bache, S. M., Müller, K., Ooms, J., Robinson, D., Seidel, D. P., Spinu, V., ..., & Yutani, H. (2019). Welcome to the tidyverse. *Journal of Open Source Software, 4*(43), 1686. https://doi.org/10.21105/joss.01686

Wilke, C. O., & Wiernik, B. M. (2022). *ggtext: Improved text rendering support for "ggplot2"* (R package version 0.1.2). https://CRAN.R-project.org/package=ggtext
